# A novel inflammation-nutrition biomarker score for predicting prognosis of patients with cancer: results from a multicenter study

**DOI:** 10.1186/s12885-022-10399-5

**Published:** 2022-12-14

**Authors:** Hailun Xie, Guotian Ruan, Lishuang Wei, Heyang Zhang, Qi Zhang, Yizhong Ge, Shiqi Lin, Mengmeng Song, Xi Zhang, Xiaoyue Liu, Xiaowei Zhang, Xiangrui Li, Kangping Zhang, Ming Yang, Meng Tang, Li Deng, Hanping Shi

**Affiliations:** 1 Department of Gastrointestinal Surgery/Department of Clinical Nutrition, Beijing Shijitan Hospital, Capital Medical University, Beijing, 100038 China; 2 Beijing International Science and Technology Cooperation Base for Cancer Metabolism and Nutrition, Beijing, 100038 China; 3 Key Laboratory of Cancer FSMP for State Market Regulation, Beijing, 100038 China; 4 Department of Geriatric Respiratory Disease Ward, the First Affiliated Hospital, Guangxi Medical University, Nanning, Guangxi China

**Keywords:** Inflammation, Malnutrition, Cancer, Prognosis

## Abstract

**Background:**

This study aimed to develop an innovative inflammation-nutrition biomarker score (INS) system to stratify the prognoses of patients with cancer.

**Methods:**

A total of 5,221 patients with cancer from multiple centers in China between June 2010 and December 2017 were enrolled in this prospective cohort study. We compared the commonly used inflammation and nutrition biomarkers and selected the most valuable to develop the novel INS system. Survival curves were assessed using the Kaplan–Meier method and the log-rank test to evaluate the difference in survival rates between groups. The Cox proportional hazards model was used to investigate the association between biomarkers and all-cause mortality.

**Results:**

As the risk stratification of INS increased (1 to 5), the rate of death for cancer patients gradually increased (25.43% vs. 37.09% vs. 44.59% vs. 56.21% vs. 61.65%, *p* < 0.001). The INS system was associated with all-cause mortality in patients with cancer. Patients with both high inflammation and nutrition risk (INS = 5) were estimated to have much worse prognosis than those with neither (HR, 2.606; 95%CI, 2.261–3.003, *p* < 0.001). Subsequently, the results of randomized internal validation also confirmed that INS system had an ideal effect in identifying adverse outcomes. In addition, the INS system could be used as a supplement to pathological stages in prognosis assessment, and had a higher predictive value in comparison with the constitute biomarkers. Patients with a high INS had less functional ability, reduced quality of life, and were at high risk of malnutrition, cachexia, and poor short-term outcomes.

**Conclusion:**

The INS system based on inflammation and nutrition biomarkers is a simple and effective prognostic stratification tool for patients with cancer, which can provide a valuable reference for clinical prognosis assessment and treatment strategy formulation.

**Supplementary Information:**

The online version contains supplementary material available at 10.1186/s12885-022-10399-5.

## Background

Inflammation is closely related to cancer, which is considered the wound that never heals. Inflammation is involved in the occurrence, proliferation, metastasis, senescence, and apoptosis of malignancy [1–3]. Germano [4] reported that inflammatory cells can create a tumor microenvironment by secreting cytokines and chemokines that help tumor cell invasion, metastasis, and angiogenesis. Ostan et al. [5] believed that inflammation triggered gene mutations or epigenetic mechanisms that promoted the development, metastasis, and progression of cancer and that interventions to reduce inflammation could reduce the risk of occurrence and progression of cancer. Some studies have reported that patients who regularly take non-steroidal anti-inflammatory drugs have significantly reduced progression and mortality from cancer [6, 7]. In clinical practice, conventional biomarkers that reflect systemic inflammation are circulating blood cells (such as neutrophils, lymphocytes, and monocytes) and acute phase proteins such as C-reactive protein (CRP). Currently, many inflammation biomarkers have been reported to be associated with the prognosis of patients with cancer [8–11]. In addition, the combination of multiple inflammation biomarkers may further improve the ability to predict prognosis.

Because the disease and its treatments threaten nutritional status, patients with cancer are at particularly high risk of malnutrition. Approximately 20% to 70% of patients with cancer experience varying degrees of malnutrition [12]. Malnutrition is another important factor leading to poor outcomes for patients with cancer. Approximately 10% to 20% of cancer deaths can be attributed to malnutrition rather than to the cancer itself [13, 14]. However, many severely malnourished people do not get the nutritional interventions that they need, with only 30% to 60% of patients with cancer at risk of malnutrition receiving nutritional support [15, 16]. Most nutrition assessment biomarkers are based on serum albumin and body weight, which have been reported to be effective in assessing the prognosis of patients with various cancers [17, 18].

Inflammation and nutritional status are both important factors affecting the clinical outcomes of patients with cancer. They are interrelated, interactive, and inseparable [19]. Inflammation is increasingly identified as an important underlying factor increasing the risk of malnutrition, which can alter the ability to use nutrients and lead to poor responses to nutritional interventions [20]. On the other hand, malnutrition can also destroy the balance between the inflammatory and anti-inflammatory in patients with cancer, leading to an overflow of inflammatory cytokines and further aggravating systemic inflammation [21–24]. The combination of inflammation and nutrition may provide new ideas for prognosis assessment, individualized risk stratification, and treatment guidance in cancer care. Therefore, this study aimed to develop an innovative inflammation-nutrition biomarker score (INS) to stratify the prognosis of patients with cancer by combining commonly used inflammation and nutrition biomarkers.

## Methods

### Study design and population

All patients were from the Investigation on Nutrition Status and Clinical Outcome of Common Cancers (INSCOC) project of China (Registration Number: ChiCTR1800020329), which prospectively enrolled patients with cancer hospitalized at more than 40 medical centers in China between June 2010 and December 2017. The inclusion criteria for this study were as follows: 1) Histopathologically confirmed cancer; 2) Complete, available clinicopathological and serological data; 3) Age 18 years or older; 4) Voluntarily participation in this study. The exclusion criteria were as follows: 1) Admission time < 24 h; 2) Severe infection and acute inflammation, 3) Continuous use of anti-inflammatory drugs within the past 6 months; 4) Acquired immunodeficiency syndrome; 5) Unwillingness or inability to participate because of cognitive impairment. This study strictly complied with the Declaration of Helsinki during the research process and was approved by the ethics committees of all participating institutions. All participants signed written informed consent to participate in this study.

### Data collection and definitions

Baseline clinicopathological variables including demographic characteristics (sex, age, height, and weight), family history of cancer, lifestyle factors (smoking and drinking), comorbidities (hypertension and diabetes), and disease information (tumor type, pathological stage, and treatment methods). Pathological stage was defined using the 8th edition of the TNM classification system of the American Joint Committee on Cancer staging system. Blood samples were collected after fasting for 10 h (before treatment). Blood tests included white blood cell (WBC), neutrophil, lymphocyte, platelet, red blood cell (RBC), hemoglobin, albumin, CRP levels, and blood glucose. In this study, biomarkers containing CRP or those previously thought to be associated with inflammation were defined as systemic inflammation biomarkers, including lymphocyte-to-CRP ratio (LCR), CRP-to-albumin ratio (CAR), modified geriatric nutrition risk index (mGNRI), neutrophil-to-lymphocyte ratio (NLR), systemic inflammation index (SII), lymphocyte-CRP score (LCS), glucose-to-lymphocyte ratio (GLR), and platelet-to-lymphocyte ratio (PLR). Biomarkers containing albumin or those previously thought to be associated with nutrition were defined as nutrition biomarkers, including the advanced lung cancer inflammation index (ALI), prognostic nutritional index (PNI), nutritional risk index (NRI), geriatric nutritional risk index (GNRI), albumin-to-globulin ratio (AGR), and the controlling nutritional status score (CONUT). These biomarkers were calculated using formulas reported in previous studies [25–28]. The formula of those indicators is presented in Table S1.

### Construction of the INS model

**Fig. 1:**
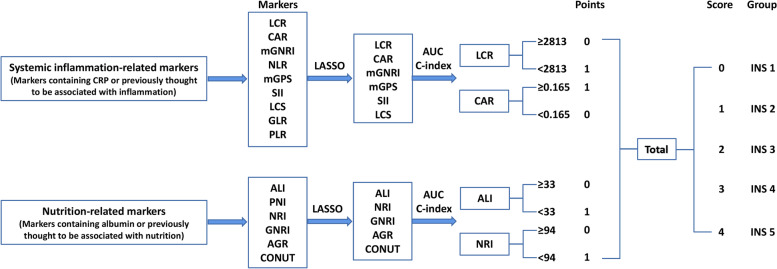
The process diagram of INS construction and risk stratification

Among these inflammation biomarkers, the LCR and CAR were the most effective biomarkers in prognostic evaluation of patients with cancer, with C-indexes of 0.652 (0.640, 0.665) and 0.649 (0.636, 0.661), respectively. Among these nutrition biomarkers, the ALI and NRI were the most effective biomarkers in prognostic evaluation of patients with cancer, with C-indexes of 0.643 (0.630, 0.655) and 0.623 (0.610, 0.636), respectively (Fig. S1). In addition, the Pearson test showed that the correlation between these biomarkers was relatively small (Fig. S2). Based on the above analysis, we selected the top two systemic inflammation and nutrition biomarkers for further analysis. The optimal stratification method determined the optimal threshold for LCR, CAR, ALI, and NRI were 2813, 0.165, 33, and 94, respectively (Fig. S3). Subsequently, we constructed a novel inflammation nutrition score (INS) system based on these inflammation and nutrition biomarkers. The process diagram of INS system construction and risk stratification was developed as follows: low LCR (< 2813), high CAR (≥ 0.165), low ALI (< 33), and low NRI (< 94) were scored as 1, and the remaining values were scored as 0. All scores were added together, and the final risk stratification was categorized into five groups (INS 1–5) according to the scores (e.g., score of 0 = INS value of 1) (Fig. 1).

### Follow-up and outcomes

Medical professionals performed follow-up evaluations (including face-to-face outpatient follow-up and telephone follow-up) until the last follow-up date (October 30, 2020) or the date of death from any cause. The primary outcome was all-cause mortality, defined as the period from the date of pathologic diagnosis to the date of death, study withdrawal, or last follow-up. Secondary outcomes included physical status, which was assessed by the Karnofsky performance status questionnaire (KPS); nutritional status, which was assessed by the Patient-generated Subjective Global Assessment (PG-SGA); quality of life, which was assessed by the European Organization for Research and Treatment of Cancer Quality of Life Questionnaire version 3.0 (EORTC QLQ-C30); cachexia, which was diagnosed according to the internationally recognized diagnostic criteria for cachexia proposed by Fearon et al. in the International Consensus on Cachexia in 2011[29]; and short-term outcome, which was defined as the patient’s outcome 90 days after treatment.

### Statistical analysis

Continuous variables were expressed as means and standard deviations (SD) or medians and interquartile differences. Categorical variables were expressed as frequencies and proportions. The Chi-square or Fisher’s exact test was used for comparison of categorical variables between groups, while the Mann–Whitney U or unpaired student’s T-test was used for continuous variables. To avoid the influence of collinearity between the various biomarkers, we used LASSO Cox regression with “glmnet” package, and Pearson’s test to eliminate biomarkers with large collinearity. Subsequently, Harrell concordance statistics (C-statistics), continuous net reclassification improvement (cNRI), integrated discrimination improvement (IDI), and the time-dependent area under the receiver operating characteristic curve (AUC) were calculated to compare the predictive capacity of biomarkers. Optimal stratification was used to solve the threshold of continuous biomarkers through log-rank statistics. Similar to previous research [30, 31], restricted cubic spline (three knots) regression with “rms” package was used to assess the relationship between continuous biomarkers and survival of patients with cancer. Survival curves were presented using the Kaplan–Meier method and log-rank test to evaluate the difference in survival rates between groups. The Cox proportional hazards model was used to investigate the association between potential biomarkers and all-cause mortality, under the model of independent effects. Meanwhile, we also assessed the dose–response relationship between the primary variable and survival to test robustness. Subsequently, we performed an internal randomization to validate the effectiveness of the model based on computer-generated random numbers in a 7:3 ratio. Univariate and multivariable logistic regression models were used to assess associations between the INS model and secondary outcomes. Model a did not adjust for any confounding factors. Model b adjusted for age, sex, BMI, TNM stage, tumor type, surgery, radiotherapy, chemotherapy, hypertension, diabetes, smoking, drinking, family history. These adjusted variables included clinicopathological information affecting the prognosis of cancer patients. All statistical analyses were performed using R, version 4.0.5 (http://www.R-project.org).

## Results

### Associations of INS system with clinicopathological characteristics

Based on the inclusion and exclusion criteria, 5,221 patients with cancer were enrolled in the study (Fig. S4), of which 3,061 (58.6%) were male and 2,160 (41.4%) were female. The mean age of the cohort was 59.41 (11.15) years. The proportion of tumor stage and tumor type among different INS groups is presented in Fig. S5. High INS was strongly associated with male, poor physical condition (advanced age, low BMI, and low KPS), unhealthy lifestyle (smoking and drinking), advanced pathological stage, poor nutritional status (low RBC, low hemoglobin, low albumin, high PG-SGA, and cachexia), high inflammation status (high neutrophils, low lymphocytes, high platelets, and high CRP). In addition, high INS was associated with adverse outcomes, including prolonged hospital stay, reduced quality of life, and poor survival (Table S2).

### Kaplan–Meier analysis of INS system in patients with cancer

**Fig. 2:**
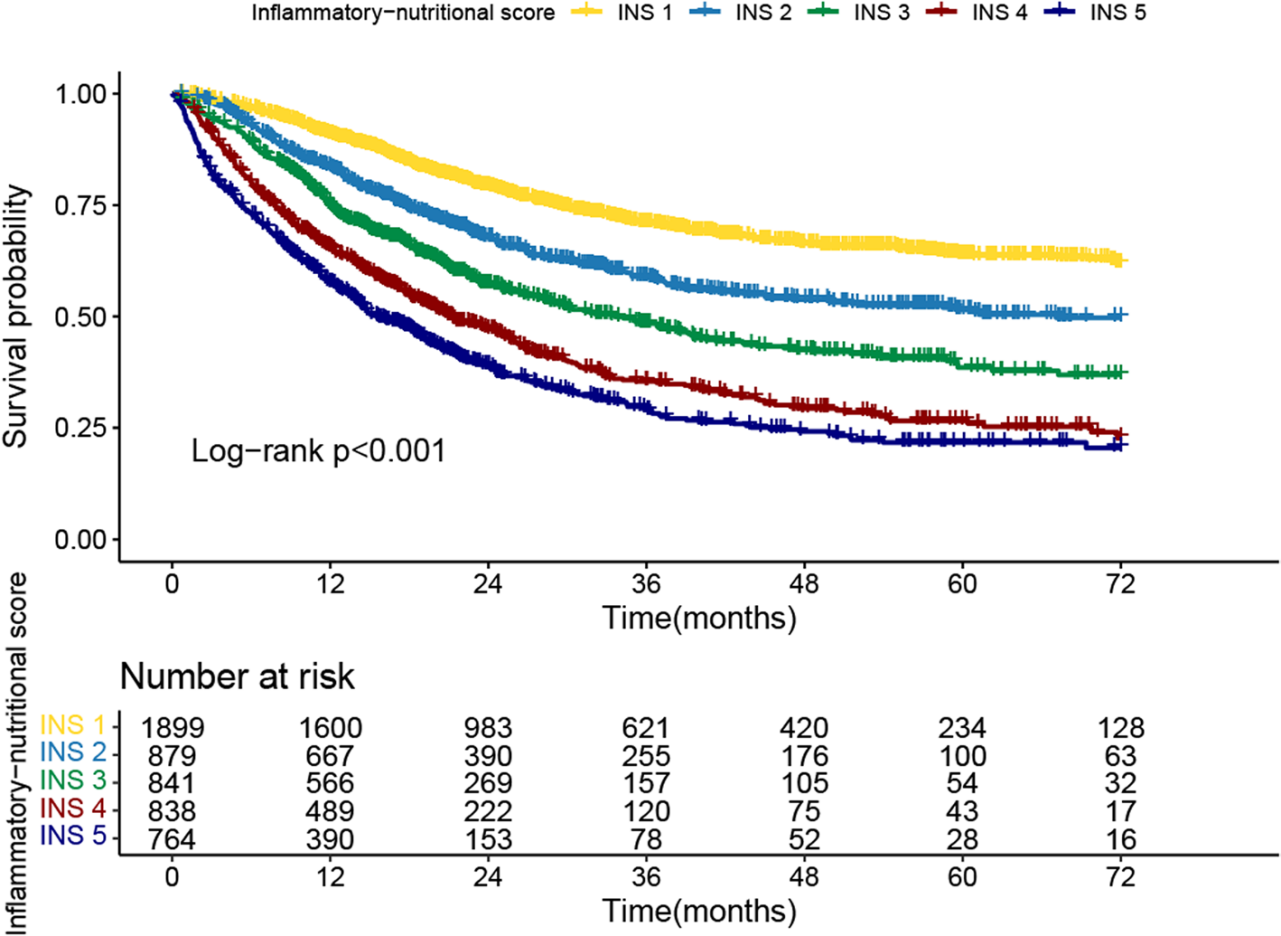
Survival curves via Kaplan–Meier analysis of inflammatory-nutritional score

Firstly, we compared the survival curves of each inflammation and nutrition marker. The results showed that low LCR, high CAR, low ALI, and low NRI were associated with poor OS of patients with cancer, with reduced survival rates of 23.2%, 22.9%, 22%, and 18.7%, respectively (Fig. S6). Meanwhile, they also effectively stratified the prognosis of patients with different pathological stages (Fig. S7A-D). Then, we compared the survival curves of each risk stratification of the INS system. As the risk stratification of INS increased, the risk of death for cancer patients gradually increased (25.43% vs. 37.09% vs. 44.59% vs. 56.21% vs. 61.65%, *p* < 0.001) (Fig. 2). Compared to patients without inflammation and nutrition risk (INS = 1), patients with extreme inflammation and nutrition risk (INS = 5) had a 36.2% higher mortality risk. It is worth noting that the prognostic stratification effect of the INS system can be clearly observed in different tumor types (gastrointestinal tumors and non-gastrointestinal tumors) (Fig. S8A). In addition, the prognosis of patients with cancer gradually decreased with risk stratification increase of the INS system in both early and advanced cancer (Fig. S8B), which indicated the INS system could be a useful complement to pathological stage in the prognosis assessment of patients with cancer.

### Relationship between INS system and all-cause mortality

We found evidence of non-linear associations (*p* value for non-linearity < 0.001) for systemic inflammation and nutrition biomarkers with all-cause mortality in patients with cancer. CAR was negatively correlated with prognosis, while other biomarkers were strongly positively correlated with prognosis (Fig. S9). We also compared the prognostic effects of these biomarkers on mortality by dividing them into four equal groups (Q1–Q4) and per SD change. The results showed that these biomarkers were factors affecting the prognosis of patients with cancer (Table S3). Meanwhile, subgroup analysis revealed that they were prognostic factors affecting patients with cancer in most subgroups (Fig. S10). In multivariable Cox regression analysis, the INS system was associated with all-cause mortality in patients with cancer. Patients with both high inflammation and nutrition risk (INS = 5) were estimated to have much worse prognoses than those with neither (HR, 2.606; 95%CI, 2.261–3.003, *p* < 0.001) (Table 1). Subgroup analysis showed that the INS system presented dose–response effects in the prognostic assessment of patients with cancer (Fig. S11A-B). In addition, the INS system had a higher prognostic predictive value in comparison with the constitute biomarkers (Table 2). It could be observed in the discriminant indicators, including C-statistics, cNRI, and IDI, that the INS model was superior to its component biomarkers in predicting the mortality of patients with cancer. For mortality risk prediction, each marker provides incremental prognostic value for pathological stages, with the full INS model providing the highest prognostic value (Table 3).

**Table 1:** Cox regression analysis of inflammation nutrition score associated with all-cause mortality

All patients				
Inflammation nutrition score	Model a	p	Model b	p
INS 1	ref			
INS 2	1.592 (1.384–1.833)	< 0.001	1.323 (1.146—1.528)	< 0.001
INS 3	2.261 (1.975–2.588)	< 0.001	1.695 (1.477—1.946)	< 0.001
INS 4	3.295 (2.901–3.743)	< 0.001	2.183 (1.912—2.493)	< 0.001
INS 5	4.175 (3.674–4.744)	< 0.001	2.606 (2.261—3.003)	< 0.001
P for trend		< 0.001		< 0.001
Validation a				
INS 1	ref			
INS 2	1.679 (1.423–1.981)	< 0.001	1.391 (1.175—1.648)	< 0.001
INS 3	2.232 (1.893–2.63)	< 0.001	1.657 (1.401—1.958)	< 0.001
INS 4	3.349 (2.875–3.901)	< 0.001	2.254 (1.925—2.64)	< 0.001
INS 5	4.173 (3.589–4.852)	< 0.001	2.575 (2.177—3.045)	< 0.001
P for trend		< 0.001		< 0.001
Validation b				
INS 1	ref			
INS 2	1.389 (1.063–1.816)	0.016	1.134 (0.862—1.493)	0.368
INS 3	2.326 (1.834–2.951)	< 0.001	1.696 (1.328—2.166)	< 0.001
INS 4	3.176 (2.519–4.005)	< 0.001	2.014 (1.579—2.569)	< 0.001
INS 5	4.198 (3.298–5.342)	< 0.001	2.714 (2.073—3.552)	< 0.001
P for trend		< 0.001		< 0.001

Model a: No adjusted

Model b: Adjusted for age, sex, BMI, TNM stage, tumor type, surgery, radiotherapy, chemotherapy, hypertension, diabetes, smoking, drinking, family history

**Table 2:** Comparative analysis of the discrimination of each biomarker for all-cause mortality in patients with cancer

Discrimination Ability	C-statistic	C-statistics		cNRI		IDI	
		Difference	*p* value	Difference	*p* value	Difference	*p* value
INS	0.663(0.651,0.674)	Ref		Ref		Ref	
LCR	0.652(0.640,0.665)	-0.044(-0.051, -0.037)	< 0.001	-0.208(-0.257, -0.052)	< 0.001	-0.022(-0.034, -0.009)	< 0.001
CAR	0.649(0.636,0.661)	-0.048(-0.055, -0.041)	< 0.001	-0.105(-0.242, -0.058)	< 0.001	-0.028(-0.040, -0.017)	< 0.001
ALI	0.643(0.630,0.655)	-0.051(-0.059, -0.041)	< 0.001	-0.273(-0.322, -0.225)	< 0.001	-0.054(-0.072, -0.039)	< 0.001
NRI	0.623(0.610,0.636)	-0.080(-0.089, -0.071)	< 0.001	-0.310(-0.348, -0.214)	< 0.001	-0.083(-0.100, -0.064)	< 0.001
mGNRI	0.639(0.627,0.652)	-0.023(-0.031, -0.014)	< 0.001	-0.126(-0.196, -0.020)	< 0.001	-0.029(-0.052, -0.007)	0.020
NLR	0.623(0.610,0.636)	-0.040(-0.051, -0.029)	< 0.001	-0.316(-0.361, -0.266)	< 0.001	-0.113(-0.134, -0.089)	< 0.001
mGPS	0.609(0.598,0.620)	-0.054(-0.063, -0.045)	< 0.001	-0.102(-0.257, -0.061)	< 0.001	-0.044(-0.061, -0.030)	< 0.001
SII	0.608(0.596,0.621)	-0.054(-0.067, -0.042)	< 0.001	-0.318(-0.359, -0.268)	< 0.001	-0.109(-0.131, -0.087)	< 0.001
LCS	0.598(0.587,0.608)	-0.065(-0.076, -0.054)	< 0.001	-0.182(-0.231, -0.072)	0.090	-0.028(-0.048, -0.009)	0.010
GLR	0.573(0.560,0.586)	-0.090(-0.104, -0.075)	< 0.001	-0.316(-0.365, -0.269)	< 0.001	-0.119(-0.139, -0.099)	< 0.001
PLR	0.570(0.556,0.583)	-0.093(-0.108, -0.079)	< 0.001	-0.320(-0.361, -0.264)	< 0.001	-0.111(-0.133, -0.087)	< 0.001
PNI	0.624(0.611,0.637)	-0.039(-0.050, -0.028)	< 0.001	-0.229(-0.291, -0.166)	< 0.001	-0.066(-0.089, -0.047)	< 0.001
GNRI	0.623(0.610,0.636)	-0.040(-0.050, -0.030)	< 0.001	-0.207(-0.270, -0.150)	< 0.001	-0.060(-0.079, -0.040)	< 0.001
AGR	0.606(0.593,0.619)	-0.057(-0.071, -0.044)	< 0.001	-0.221(-0.278, -0.176)	< 0.001	-0.062(-0.086, -0.039)	< 0.001
CONUT	0.599(0.586,0.612)	-0.064(-0.076, -0.051)	< 0.001	-0.271(-0.327, -0.223)	< 0.001	-0.084(-0.104, -0.068)	< 0.001

*cNRI* Continuous net reclassification improvement, *IDI* Integrated discrimination improvement

**Table 3:** Model performance after the addition of each biomarker to the TNM stage for predicting all-cause mortality

Discrimination Ability	C-statistic	*p* value	cNRI		IDI	
			Difference	*p* value	Difference	*p* value
TNM stage	0.664(0.653,0.674)	< 0.001	Ref		Ref	
TNM stage + INS	0.717(0.706,0.728)	< 0.001	0.273(0.225,0.323)	< 0.001	0.035(0.022,0.048)	< 0.001
TNM stage + LCR	0.694(0.684,0.705)	< 0.001	0.306(0.259,0.353)	< 0.001	0.030(0.019,0.042)	< 0.001
TNM stage + CAR	0.693(0.683,0.704)	< 0.001	0.293(0.247,0.335)	< 0.001	0.028(0.018,0.040)	< 0.001
TNM stage + ALI	0.693(0.682,0.704)	< 0.001	0.230(0.184,0.278)	< 0.001	0.014(0.005,0.023)	< 0.001
TNM stage + NRI	0.688(0.677,0.699)	< 0.001	0.144(0.096,0.183)	< 0.001	0.010(0.002,0.018)	0.014
TNM stage + mGNRI	0.700(0.689,0.712)	< 0.001	0.227(0.172,0.283)	< 0.001	0.036(0.022,0.052)	< 0.001
TNM stage + NLR	0.696(0.685,0.708)	< 0.001	0.127(0.024,0.211)	< 0.001	0.003(0.001,0.005)	< 0.001
TNM stage + mGPS	0.697(0.686,0.709)	< 0.001	0.266(0.225,0.300)	< 0.001	0.023(0.014,0.031)	< 0.001
TNM stage + SII	0.691(0.680,0.703)	< 0.001	0.079(-0.064,0.158)	0.189	0.004(0.001,0.007)	0.010
TNM stage + LCS	0.688(0.677,0.699)	< 0.001	0.336(0.278,0.382)	0.010	0.030(0.020,0.040)	< 0.001
TNM stage + GLR	0.683(0.671,0.694)	< 0.001	0.068(-0.015,0.193)	0.080	0.000(0.000,0.001)	0.109
TNM stage + PLR	0.680(0.669,0.692)	< 0.001	0.023(-0.339,0.078)	0.507	0.001(-0.001,0.004)	0.259
TNM stage + PNI	0.700(0.688,0.711)	< 0.001	0.084(0.025,0.136)	0.010	0.012(0.003,0.021)	< 0.001
TNM stage + GNRI	0.702(0.690,0.713)	< 0.001	0.124(0.063,0.166)	< 0.001	0.019(0.009,0.029)	< 0.001
TNM stage + AGR	0.693(0.681,0.704)	< 0.001	0.120(0.060,0.162)	< 0.001	0.019(0.009,0.031)	< 0.001
TNM stage + CONUT	0.694(0.683,0.705)	< 0.001	0.016(-0.030,0.062)	0.338	0.009(0.004,0.015)	0.010

*cNRI* Continuous net reclassification improvement, *IDI* Integrated discrimination improvement

### Randomized internal validation of the INS system

**Fig. 3:**
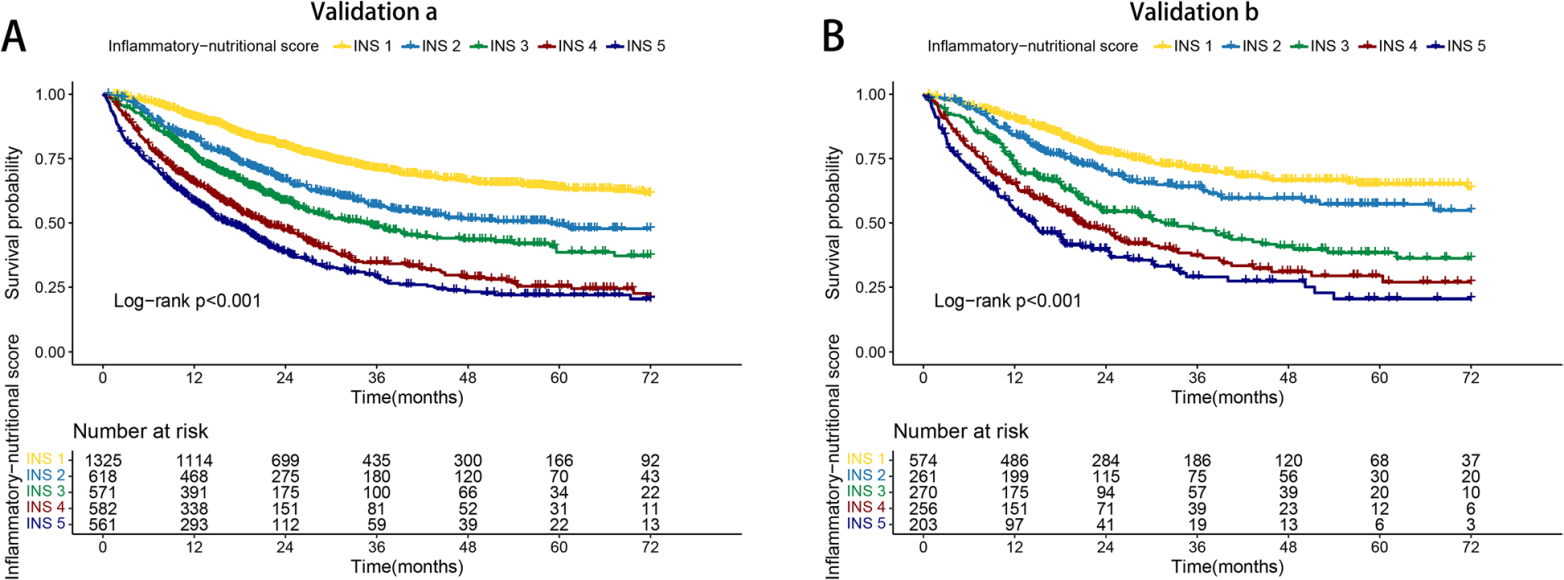
Internal validation of inflammatory-nutritional score based different tumor types and pathological stages. Notes: **A**, Validation cohort a; **B**, Validation cohort b

To validate the effectiveness of the novel INS system, we proceeded to a randomized internal validation. We randomly assigned the total population to validation cohort a (3,657 cases) and validation cohort b (1,564 cases) groups. In validation cohort a, the prognosis of patients with high INS was worse than that of patients with low INS. Patients with INS 5 had the worst prognosis, while patients with INS 1 had the best prognosis (25.51% vs. 61.85%, *p* < 0.001) (Fig. 3A). Similarly, INS was an effective prognostic stratification tool for patients with cancer in validation cohort b. The survival rate of patients with different INS showed a stepwise decline (61.08% vs. 55.86% vs. 47.41% vs. 32.57% vs. 25.26%, *p* < 0.001) (Fig. 3B). In addition, the results of multivariable Cox regression analysis showed that the INS system was a risk factor affecting the prognosis of patients with cancer both in validation cohorts a and b. Compared with low-risk patients (INS = 1), high-risk patients (INS = 5) had a 1.575 times higher risk of adverse prognosis in validation cohort a and 1.714 times in validation cohort b (Table 1).

### Relationship between the INS system and secondary outcomes

Finally, we further explored the effects of the INS system on several secondary outcomes in patients with cancer, including life function, quality of life, malnutrition, cachexia, and short-term outcomes. Multivariable logistic regression modeling indicated that the INS system was a factor affecting life function, quality of life, malnutrition, cachexia, and short-term outcomes in patients with cancer. Compared with patients with a low INS, patients with a high INS had poorer life function, reduced quality of life, poor short-term outcomes, and were at higher risk of malnutrition and cachexia (Table S4).

## Discussion

As far as we know, this study is the first to develop a novel system to predict the prognosis of patients with cancer by combining inflammation and nutrition biomarkers. Combining the advantages of these biomarkers, the INS can effectively stratify the prognosis of patients with cancer. The host-tumor interaction has a profound impact on the general condition of patients, including daily activities and nutritional status. We further found that high INS was associated with reduced life function, malnutrition, cachexia, poor quality of life, and poor short-term prognosis. In addition, this study takes the lead in comprehensively and systematically comparing the prognostic abilities of commonly used inflammatory and nutrition biomarkers in patients with cancer.

In recent years, many inflammation and nutrition biomarkers have been developed and proven to be effective in predicting the prognosis of cancer [18, 32]. Peripheral blood cell biomarkers are a good choice for establishing prognostic models based on inflammatory and nutrition biomarkers because they are convenient, reproducible, and low-cost. However, due to the heterogeneity of different studies, the optimal prognostic biomarkers for patients with cancer are still unclear, which leads to their limited clinical value. A meta-analysis by Jiang et al. [25] comprehensively compared the value of commonly used inflammation and nutrition biomarkers in predicting the prognosis of esophageal cancer, which might provide further inspiration for comprehensive comparison of prognostic biomarkers.

In this study, we compared 15 biomarkers commonly used for prognostic evaluation of patients with cancer. The results showed that LCR was the optimal inflammation marker to evaluate the prognosis of patients with cancer. This indicated that more attention should be paid to CRP-based inflammation biomarkers, such as LCR and CAR, when evaluating the inflammatory status of patients with cancer. When evaluating the prognostic value of nutritional biomarkers, ALI showed the optimal prognostic prediction performance, which may be explained by its combination of albumin and body weight. This suggested that more attention should be paid to biomarkers related to albumin and body weight, such as ALI, NRI, and GNRI, when assessing the nutritional status of patients with cancer. Notably, a single marker usually has certain limitations, which cannot comprehensively reflect the patient’s inflammation and nutritional status. Therefore, the combination of multiple biomarkers may be an effective means to improve prognosis prediction. However, the collinearity between different biomarkers can lead to instability of the marker combination. Therefore, we used LASSO Cox regression to effectively select valuable variables, and used Pearson’s test to detect their correlation.

At present, pathological stage is the most commonly used tool to evaluate prognosis and guide treatment of cancer, but there are still differences in survival of patients with cancer with the same pathological stage. Therefore, it is necessary to use effective, objective biomarkers for additional evaluation. If the only focus is pathological stage, the comprehensive evaluation of disease progression will be affected. Therefore, it is necessary to use effective and objective biomarkers for additional assessment of the prognosis of patients with cancer. In our study, we found that the INS system could be used as an effective complement to pathological staging in the prognostic assessment of patients with cancer. Interestingly, when the tumor progressed to an advanced stage, the inflammation and nutritional status of the patients could still distinguish patient’s prognosis, but the degree of discrimination was minimal. This may be because advanced patients are at increased risk of high inflammatory load and nutrient depletion that cannot be corrected by conventional methods. These findings suggested the importance of early assessment and correction of the inflammation and nutritional status of patients with cancer.

Inflammation and nutritional status are important factors affecting the prognosis of patients with cancer. In this study, we developed a novel INS system that integrates inflammation and nutrition biomarkers and found it is a simple and effective tool for assessing the prognosis of patients with cancer. However, this study still has some limitations that should be noted. This study was a multi-center, prospective, cohort study with randomized internal validation, which provided a solid foundation for the reliability of this study. However, larger samples and more centers are still needed for external validation in the future. All patients were from medical institutions in China, so whether the INS system can be applied to populations in other countries remains to be explored. Although we examined subgroups of patients with gastrointestinal tumors, a more detailed subgroup analysis was not performed due to the relatively small numbers of other specific types of tumor. In addition, we excluded many patients with CRP deficiency, which might lead to potential selection bias.

## Conclusions

In this study, a novel INS system combining inflammation and nutrition biomarkers was developed for the first time and proved to be a simple and effective prognostic stratification tool for patients with cancer, which could provide a valuable reference for clinical prognosis assessment and treatment strategy formulation.

## Supplementary Information

**Additional file 1: Table S1.** The formula of inflammation and malnutrition biomarkers. **Table S2.** The descriptive characteristics of patients with cancer. **Table S3.** Cox regression analysis of systemic inflammation-related and nutrition-related markers associated with overall survival. **Table S4.** Logistic regression analysis of inflammation nutrition score associated with secondary outcome. **Figure S1.** The area under the receiver operating characteristic curve of systemic inflammation-related and nutrition-related markers. **Figure S2.** Pearson’s test and LASSO Cox regression model. **Figure S3.** The optimal threshold of systemic inflammation-related and nutrition-related markers. **Figure S4.** Study design. **Figure S5.** The proportion of tumor stages and tumor types among different INS groups. **Figure S6.** Kaplan-Meier curve of systemic inflammation-related and nutrition-related markers in patients with cancer. **Figure S7.** Stratified survival analysis of systemic inflammation-related and nutrition-related markers based on pathological stages. **Figure S8.** Kaplan-Meier survival curve of inflammation-malnutrition biomarker score based different tumor types and pathological stages. **Figure S9.** The association between systemic inflammation-related and nutrition-related markers and all-cause mortality in patients with cancer. **Figure S10.** The association between systemic inflammation-related and nutrition-related markers and hazard risk of overall survival in various subgroups. **Figure S11.** Dose-response effects of inflammatory-nutritional score based on subgroup.

## Data Availability

All data needed to evaluate the conclusions of the study are presented in this paper and/or the Supplementary Materials. Additional data related to this study is available upon request to authors/corresponding author.
