# Supplementary material for: A novel inflammation-nutrition biomarker score for predicting prognosis of patients with cancer: results from a multicenter study
Source: BMC Cancer. 2022 Dec 14;22:1311. doi: 10.1186/s12885-022-10399-5 (PMC9749281; doi:10.1186/s12885-022-10399-5)
Supplement: Supplementary file 1 — Additional file 1: Table S1. The formula of inflammation and malnutrition biomarkers. Table S2. The descriptive characteristics of patients with cancer. Table S3. Cox regression analysis of systemic inflammation-related and nutrition-related markers associated with overall survival. Table S4. Logistic regression analysis of inflammation nutrition score associated with secondary outcome. Figure S1. The area under the receiver operating characteristic curve of systemic inflammation-related and nutrition-related markers. Figure S2. Pearson’s test and LASSO Cox regression model. Figure S3. The optimal threshold of systemic inflammation-related and nutrition-related markers. Figure S4. Study design. Figure S5. The proportion of tumor stages and tumor types among different INS groups. Figure S6. Kaplan-Meier curve of systemic inflammation-related and nutrition-related markers in patients with cancer. Figure S7. Stratified survival analysis of systemic inflammation-related and nutrition-related markers based on pathological stages. Figure S8. Kaplan-Meier survival curve of inflammation-malnutrition biomarker score based different tumor types and pathological stages. Figure S9. The association between systemic inflammation-related and nutrition-related markers and all-cause mortality in patients with cancer. Figure S10. The association between systemic inflammation-related and nutrition-related markers and hazard risk of overall survival in various subgroups. Figure S11. Dose-response effects of inflammatory-nutritional score based on subgroup. [file 12885_2022_10399_MOESM1_ESM.docx]

**Table S1.** The formula of inflammation and malnutrition biomarkers.

| Indicators | The formula |
| --- | --- |
| INS | Low LCR (<2813), high CAR (≥0.165), low ALI (<33), and low NRI (<94) were scored as 1, and the remaining values were scored as 0. All scores were added together, and the final risk stratification was categorized into five groups (INS 1-5) according to the scores |
| LCR | 10,000×lymphocyte(10^9/l)/CRP(mg/l) |
| CAR | C-reactive protein(mg/l)/albumin(g/l) |
| ALI | BMI×albumin / NLR (neutrophil/lymphocyte ratio) |
| NRI | 1.519× albumin(g/L)+ 41.7×(body weight/ideal body weight). We set BW/IBW =1 when BW exceeded IBW. Ideal body weight was calculated using the Lorentz formula, as follows: Male, IBW = height - 100 - (height -150)/ 4;Female,IBW = height -100-(height -150)/2.5. |
| mGNRI | 14.89/CRP(mg/L) + 41.7 × (body weight / ideal body weight). We set BW/IBW =1 when BW exceeded IBW. Ideal body weight was calculated using the Lorentz formula, as follows: Male, IBW = height -100 -(height -150) / 4; Female, IBW = height -100-(height -150) / 2.5. |
| NLR | Neutrophil/lymphocyte ratio |
| mGPS | C-reactive protein≤10mg/L and albumin ≥35g/L: 0 score  C-reactive protein≤10mg/L and albumin < 35g/L: 0 score  C-reactive protein > 10mg/L: 1 score  C-reactive protein >10mg/L and albumin < 35g/L: 2 score |
| SII | Neutrophil×platelet/lymphocyte ratio |
| LCS | Lymphocyte count ≥1×10^9/L and C-reactive protein ≤ 3 mg/L: 0 score;  Lymphocyte count ≤1×10^9/L or C-reactive protein ≥ 3 mg/L: 1 score;  Lymphocyte count ≤1×10^9/L and C-reactive protein ≥ 3 mg/L: 2 score |
| GLR | Glucose(mmol/l) / lymphocyte (10^9/L) |
| PLR | Platelet / lymphocyte |
| PNI | Albumin (g/L) + 5 × lymphocyte（10^9/L） |
| GNRI | 1.489 × albumin (g/L)+ 41.7 × (body weight / ideal body weight). We set BW / IBW =1 when BW exceeded IBW. Ideal body weight was calculated using the Lorentz formula, as follows: Male, IBW = height - 100 - (height -150) / 4; Female, IBW = height - 100 - (height -150) / 2.5. |
| AGR | Albumin (g/L) / globulin (g/L) |
| CONUT | (1)  Albumin(g/L), ≥35 0 score;  Albumin(g/L), 30-34.9 2 score;  Albumin(g/L), 25-29.9 4 score;  Albumin(g/L), <25 6 score;  (2)  Lymphocyte(×10^9/L), >1.6 0 score;  Lymphocyte(×10^9/L), 1.2-1.599 1 score;  Lymphocyte(×10^9/L), 0.8-1.199 2 score;  Lymphocyte(×10^9/L), <0.8 3 score;  (3)  Total cholesterol(mmol/L), ≥4.662 0 score;  Total cholesterol(mmol/L), 3.626-4.662 1 score;  Total cholesterol(mmol/L), 2.59-3.626 2 score;  Total cholesterol(mmol/L), <2.59 3 score; |

**Table S2.** The descriptive characteristics of patients with cancer.

| **Characteristic** | Overall | INS system | | | | | |
| --- | --- | --- | --- | --- | --- | --- | --- |
|  | n=5221 | INS 1 (n=1899) | INS 2 (n=879) | INS 3 (n=841) | INS 4 (n=838) | INS 5 (n=764) | p |
| Sex, male, n (%) | 3061 (58.6) | 997 (52.5) | 478 (54.4) | 520 (61.8) | 546 (65.2) | 520 (68.1) | <0.001 |
| Age, yearsc | 59.41 (11.15) | 57.51 (10.80) | 58.94 (11.34) | 59.63 (11.05) | 61.19 (10.87) | 62.51 (11.22) | <0.001 |
| BMI, kg/m^2^, mean (SD) | 22.57 (3.51) | 23.51 (4.19) | 21.64 (4.26) | 22.84 (5.02) | 22.48 (4.05) | 19.89 (4.22) | <0.001 |
| Hypertension, yes, n (%) | 1085 (20.8) | 341 (18.0) | 155 (17.6) | 206 (24.5) | 225 (26.8) | 158 (20.7) | <0.001 |
| Diabetes, yes, n (%) | 530 (10.2) | 162 ( 8.5) | 71 ( 8.1) | 94 (11.2) | 112 (13.4) | 91 (11.9) | <0.001 |
| Smoking yes, n (%) | 2431 (46.6) | 779 (41.0) | 375 (42.7) | 417 (49.6) | 426 (50.8) | 434 (56.8) | <0.001 |
| Drinking, yes, n (%) | 1166 (22.3) | 369 (19.4) | 181 (20.6) | 214 (25.4) | 208 (24.8) | 194 (25.4) | <0.001 |
| Family history, yes, n (%) | 871 (16.7) | 351 (18.5) | 134 (15.2) | 152 (18.1) | 136 (16.2) | 98 (12.8) | 0.004 |
| Lung cancer, yes, n (%) | 1772 (33.9) | 576 (30.3) | 266 (30.3) | 325 (38.6) | 333 (39.7) | 272 (35.6) | <0.001 |
| Gastric cancer, yes, n (%) | 434 (8.3) | 50 ( 2.6) | 64 ( 7.3) | 85 (10.1) | 119 (14.2) | 116 (15.2) | <0.001 |
| Esophagus cancer, yes, n (%) | 315 (6.0) | 79 ( 4.2) | 58 ( 6.6) | 57 ( 6.8) | 62 ( 7.4) | 59 ( 7.7) | 0.001 |
| Hepatic-biliary cancer, yes, n (%) | 586 (11.2) | 289 (15.2) | 137 (15.6) | 53 ( 6.3) | 48 ( 5.7) | 59 ( 7.7) | <0.001 |
| Pancreatic cancer, yes, n (%) | 137 (2.6) | 27 ( 1.4) | 18 ( 2.0) | 31 ( 3.7) | 36 ( 4.3) | 25 ( 3.3) | <0.001 |
| Colorectal cancer, yes, n (%) | 915 (17.5) | 404 (21.3) | 144 (16.4) | 143 (17.0) | 114 (13.6) | 110 (14.4) | <0.001 |
| Gynecological cancer, yes, n (%) | 175 (3.4) | 54 ( 2.8) | 33 ( 3.8) | 30 ( 3.6) | 32 ( 3.8) | 26 ( 3.4) | 0.619 |
| Urologic cancer, yes, n (%) | 107 (2.0) | 28 ( 1.5) | 21 ( 2.4) | 22 ( 2.6) | 10 ( 1.2) | 26 ( 3.4) | 0.005 |
| Nasopharynx cancer, yes, n (%) | 120 (2.3) | 57 ( 3.0) | 28 ( 3.2) | 12 ( 1.4) | 16 ( 1.9) | 7 ( 0.9) | 0.002 |
| Breast cancer, yes, n (%) | 464 (8.9) | 282 (14.8) | 83 ( 9.4) | 53 ( 6.3) | 25 ( 3.0) | 21 ( 2.7) | <0.001 |
| Other cancer, yes, n (%) | 196 (3.8) | 53 ( 2.8) | 27 ( 3.1) | 30 ( 3.6) | 43 ( 5.1) | 43 ( 5.6) | 0.001 |
| TNM stage, n (%) |  |  |  |  |  |  | <0.001 |
| Stage I | 441 (8.4) | 242 (12.7) | 79 ( 9.0) | 59 ( 7.0) | 36 ( 4.3) | 25 ( 3.3) |  |
| Stage II | 943 (18.1) | 458 (24.1) | 177 (20.1) | 131 (15.6) | 97 (11.6) | 80 (10.5) |  |
| Stage III | 1413 (27.1) | 601 (31.6) | 253 (28.8) | 210 (25.0) | 194 (23.2) | 155 (20.3) |  |
| Stage IV | 2424 (46.4) | 598 (31.5) | 370 (42.1) | 441 (52.4) | 511 (61.0) | 504 (66.0) |  |
| Surgery, yes, n (%) | 2596 (49.7) | 1150 (60.6) | 472 (53.7) | 382 (45.4) | 320 (38.2) | 272 (35.6) | <0.001 |
| Radiotherapy, yes, n (%) | 581 (11.1) | 176 ( 9.3) | 112 (12.7) | 97 (11.5) | 103 (12.3) | 93 (12.2) | 0.026 |
| Chemotherapy, yes, n (%) | 3312 (63.4) | 1260 (66.4) | 548 (62.3) | 574 (68.3) | 513 (61.2) | 417 (54.6) | <0.001 |
| WBC, median (IQR) | 5.90 (2.99) | 5.25 (2.1) | 5.80 (3.07) | 5.90 (2.87) | 7.00 (3.66) | 7.51 (4.29) | <0.001 |
| Neutrophil, mean (SD) | 3.70 (2.6) | 2.94 (1.48) | 3.75 (2.7) | 3.70 (2.26) | 5.03 (3.2) | 5.50 (3.63) | <0.001 |
| Lymphocyte, mean (SD) | 1.50 (0.81) | 1.75 (0.67) | 1.40 (0.63) | 1.50 (0.90) | 1.27 (0.75) | 1.14 (0.73) | <0.001 |
| Platelet, median (IQR) | 4.24 (0.82) | 212.00 (92.00) | 210.00 (101.5) | 223.00 (110.00) | 237.00 (123.00) | 254.00 (151.5) | <0.001 |
| RBC, median (IQR) | 222.00 (110.00) | 4.41 (0.71) | 4.23 (0.81) | 4.20 (0.81) | 4.17 (0.85) | 3.80 (0.92) | <0.001 |
| Hemoglobin, median (IQR) | 126.00 (28.00) | 132.00 (22.00) | 126.00 (26.00) | 125.00 (26.00) | 123.00 (28.75) | 109.00 (31.00) | <0.001 |
| Albumin, mean (SD) | 39.50 (6.4) | 41.60 (4.6) | 40.00 (5.7) | 39.30 (5.6) | 38.20 (4.98) | 32.80 (4.92) | <0.001 |
| CRP, median (IQR) | 3.71 (13.2) | 2.97 (2.19) | 3.02 (2.37) | 8.45 (16.1) | 18.05 (31.0) | 37.00 (59.97) | <0.001 |
| KPS, mean (SD) | 90.00 (10.00) | 89.14 (8.54) | 86.89 (10.24) | 85.43 (12.10) | 82.43 (13.15) | 77.58 (16.82) | <0.001 |
| PG.SGA, median (IQR) | 5.00 (7.00) | 3.00 (4.00) | 4.00 (6.00) | 5.00 (7.00) | 7.00 (7.00) | 9.00 (9.00) | <0.001 |
| Cachexia, yes (%) | 1719 (32.9) | 424 (22.3) | 267 (30.4) | 268 (31.9) | 330 (39.4) | 430 (56.3) | <0.001 |
| EORTC QLQ-C30, median (IQR) | 47.00 (11.00) | 46.00 (9.00) | 46.00 (10.50) | 47.00 (11.00) | 49.00 (13.00) | 53.00 (16.00) | <0.001 |
| Short-term outcome, death, n (%) | 318 (6.1) | 27 ( 1.4) | 18 ( 2.0) | 48 ( 5.7) | 84 (10.0) | 141 (18.5) | <0.001 |
| Long-term outcome, death, n (%) | 2149 (41.2) | 483 (25.4) | 326 (37.1) | 375 (44.6) | 471 (56.2) | 471 (61.6) | <0.001 |
| LOS, median (IQR) | 13.50 (13.13) | 9.00 (8.50) | 10.00 (9.00) | 10.00 (9.00) | 11.00 (10.00) | 12.00 (10.00) | <0.001 |

**Table S3.** Cox regression analysis of systemic inflammation-related and nutrition-related markers associated with overall survival.

| Systemic inflammation-related markers | | | | |
| --- | --- | --- | --- | --- |
| LCR | Model a | | Model b | |
| Continuous (per SD) | 0.674(0.619,0.735) | <0.001 | 0.79(0.731,0.853) | <0.001 |
| Cutoff value | 0.404(0.371,0.441) | <0.001 | 0.56(0.512,0.613) | <0.001 |
| Quartiles |  |  |  |  |
| Q1 | ref |  | ref |  |
| Q2 | 0.653(0.587,0.727) | <0.001 | 0.801(0.718,0.892) | <0.001 |
| Q3 | 0.388(0.344,0.438) | <0.001 | 0.553(0.489,0.627) | <0.001 |
| Q4 | 0.290(0.256,0.328) | <0.001 | 0.469(0.412,0.533) | <0.001 |
| p for trend |  | <0.001 |  | <0.001 |
| CAR |  |  |  |  |
| Continuous (per SD) | 1.268 (1.224,1.313) | <0.001 | 1.169(1.125,1.214) | <0.001 |
| Cutoff value | 2.407 (2.21,2.621) | <0.001 | 1.721(1.575,1.881) | <0.001 |
| Quartiles |  |  |  |  |
| Q1 | ref |  | ref |  |
| Q2 | 1.286 (1.118,1.48) | <0.001 | 1.156(1.004,1.331) | 0.045 |
| Q3 | 2.132 (1.878,2.42) | <0.001 | 1.682(1.478,1.914) | <0.001 |
| Q4 | 3.272 (2.897,3.696) | <0.001 | 2.061(1.816,2.338) | <0.001 |
| p for trend |  | <0.001 |  | <0.001 |
| Nutritional-related markers | | | | |
| ALI |  |  |  |  |
| Continuous (per SD) | 0.650 (0.612,0.691) | <0.001 | 0.811 (0.764,0.861) | <0.001 |
| Cutoff value | 0.446 (0.410,0.487) | <0.001 | 0.626 (0.570,0.686) | <0.001 |
| Quartiles |  |  |  |  |
| Q1 | ref |  | ref |  |
| Q2 | 0.624 (0.560,0.695) | <0.001 | 0.735 (0.659,0.820) | <0.001 |
| Q3 | 0.398 (0.353,0.448) | <0.001 | 0.560 (0.495,0.635) | <0.001 |
| Q4 | 0.323 (0.285,0.366) | <0.001 | 0.525 (0.458,0.601) | <0.001 |
| p for trend |  | <0.001 |  | <0.001 |
| NRI |  |  |  |  |
| Continuous (per SD) | 0.698 (0.672,0.726) | <0.001 | 0.804 (0.768,0.843) | <0.001 |
| Cutoff value | 0.495 (0.453,0.541) | <0.001 | 0.601 (0.545,0.664) | <0.001 |
| Quartiles |  |  |  |  |
| Q1 | ref |  | ref |  |
| Q2 | 0.660 (0.592,0.737) | <0.001 | 0.700 (0.624,0.784) | <0.001 |
| Q3 | 0.504 (0.448,0.566) | <0.001 | 0.590 (0.520,0.670) | <0.001 |
| Q4 | 0.371 (0.328,0.420) | <0.001 | 0.492 (0.428,0.565) | <0.001 |
| p for trend |  | <0.001 |  | <0.001 |

Notes:

Model a: No adjusted.

Model b: Adjusted for age, sex, BMI, TNM stage, tumor type, surgery, radiotherapy, chemotherapy, hypertension, diabetes, smoking, drinking, family history.

**Table S4.** Logistic regression analysis of inflammation nutrition score associated with secondary outcome.

| Life function (KPS) | | | | |
| --- | --- | --- | --- | --- |
| INS | Model a | | Model b | |
| INS 1 | ref |  | ref |  |
| INS 2 | 1.931(1.431,2.605) | < 0.001 | 1.404(1.027,1.920) | 0.033 |
| INS 3 | 2.934(2.22,3.878) | < 0.001 | 2.168(1.614,2.911) | < 0.001 |
| INS 4 | 5.114(3.95,6.619) | < 0.001 | 3.424(2.597,4.514) | < 0.001 |
| INS 5 | 8.055(6.259,10.366) | < 0.001 | 4.174(3.137,5.552) | < 0.001 |
| Malnutrition (PGSGA) | | | | |
| INS 1 | ref |  | ref |  |
| INS 2 | 1.754(1.492,2.062) | < 0.001 | 1.394(1.172,1.658) | < 0.001 |
| INS 3 | 1.986(1.683,2.344) | < 0.001 | 1.819(1.522,2.175) | < 0.001 |
| INS 4 | 3.360(2.815,4.010) | < 0.001 | 2.956(2.440,3.580) | < 0.001 |
| INS 5 | 8.018(6.398,10.048) | < 0.001 | 5.199(4.074,6.635) | < 0.001 |
| Quality of life (EORTC QLQ-C30) | | | | |
| INS 1 | ref |  | ref |  |
| INS 2 | 0.991(0.784,1.254) | 0.942 | 0.944(0.740,1.203) | 0.640 |
| INS 3 | 1.193(0.929,1.532) | 0.168 | 1.070(0.826,1.386) | 0.608 |
| INS 4 | 1.380(1.062,1.792) | 0.016 | 1.217(0.923,1.603) | 0.164 |
| INS 5 | 2.577(1.844,3.603) | < 0.001 | 2.248(1.570,3.220) | <0.001 |
| Cachexia | | | | |
| INS 1 | ref |  | ref |  |
| INS 2 | 1.518(1.268,1.817) | < 0.001 | 1.078(0.887,1.310) | 0.452 |
| INS 3 | 1.627(1.358,1.950) | < 0.001 | 1.450(1.188,1.769) | < 0.001 |
| INS 4 | 2.260(1.896,2.694) | < 0.001 | 2.044(1.680,2.487) | < 0.001 |
| INS 5 | 4.479(3.744,5.357) | < 0.001 | 2.614(2.122,3.220) | < 0.001 |
| Short-term outcomes | | | | |
| INS 1 | ref |  | ref |  |
| INS 2 | 1.449(0.794,2.646) | 0.227 | 1.096(0.595,2.018) | 0.769 |
| INS 3 | 4.197(2.600,6.774) | < 0.001 | 2.898(1.775,4.732) | < 0.001 |
| INS 4 | 7.724(4.966,12.014) | < 0.001 | 4.609(2.918,7.280) | < 0.001 |
| INS 5 | 15.692(10.294,23.920) | < 0.001 | 7.821(4.961,12.331) | < 0.001 |

**Figure. S1.** The area under the receiver operating characteristic curve of systemic inflammation-related and nutrition-related markers.


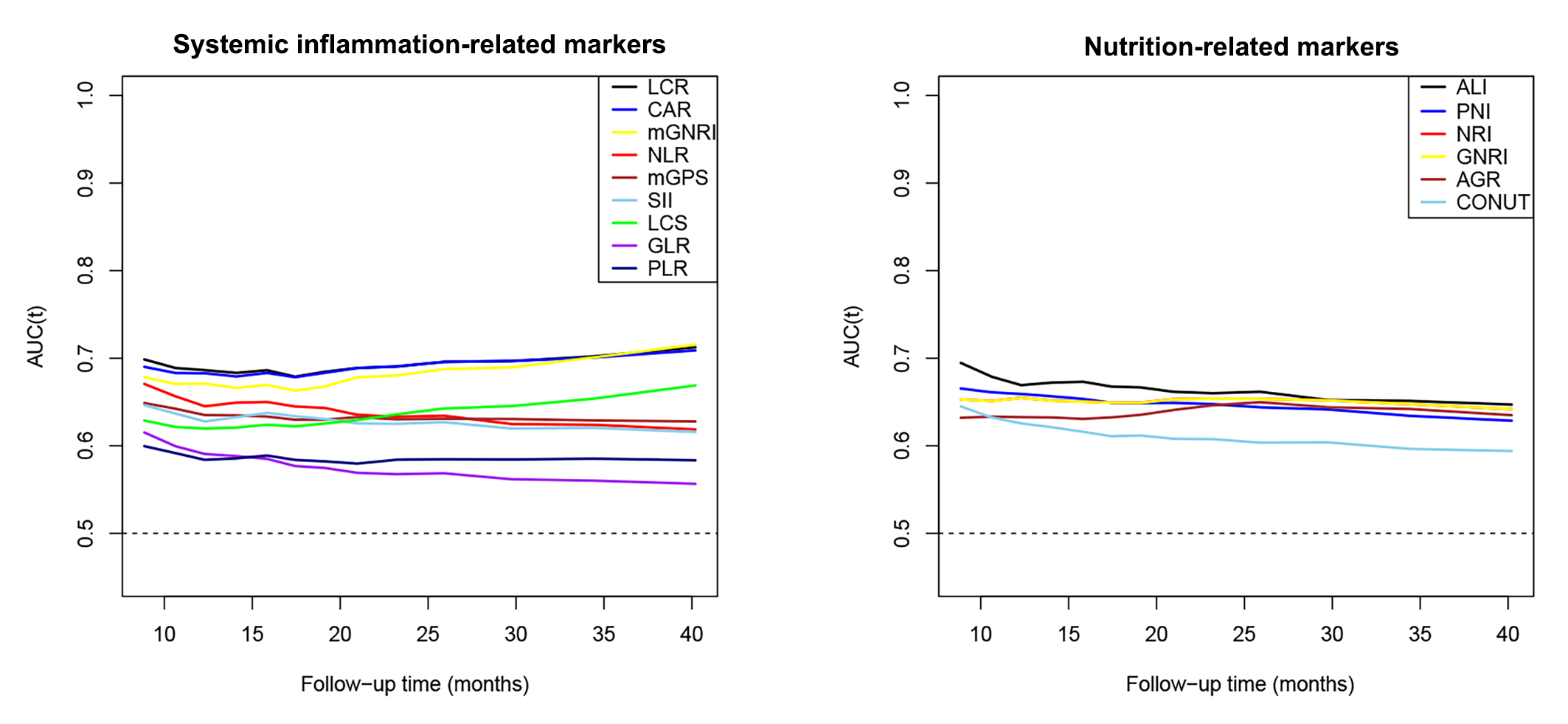


**Figure. S2.** Pearson’s test and LASSO Cox regression model.


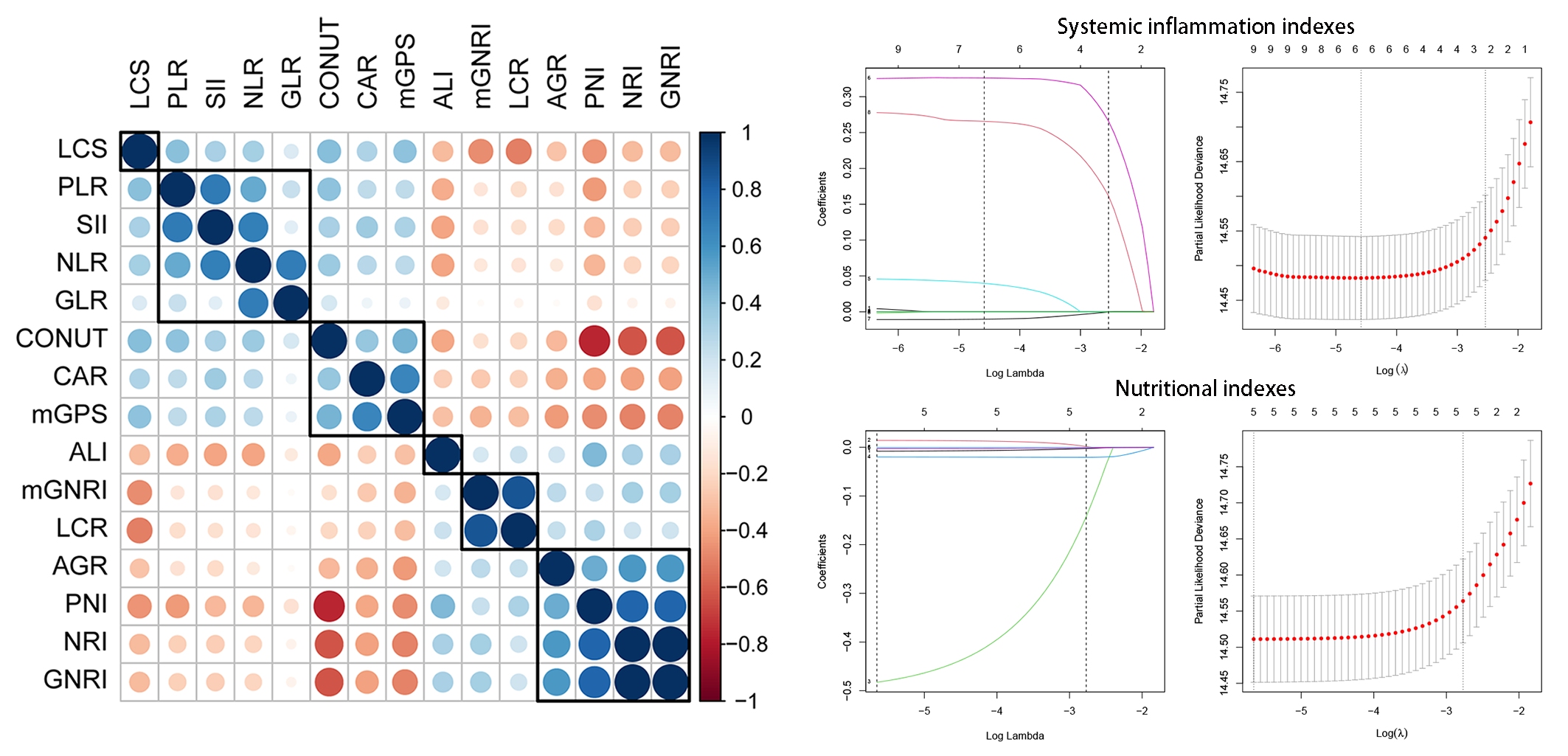


**Figure. S3.** The optimal threshold of systemic inflammation-related and nutrition-related markers.


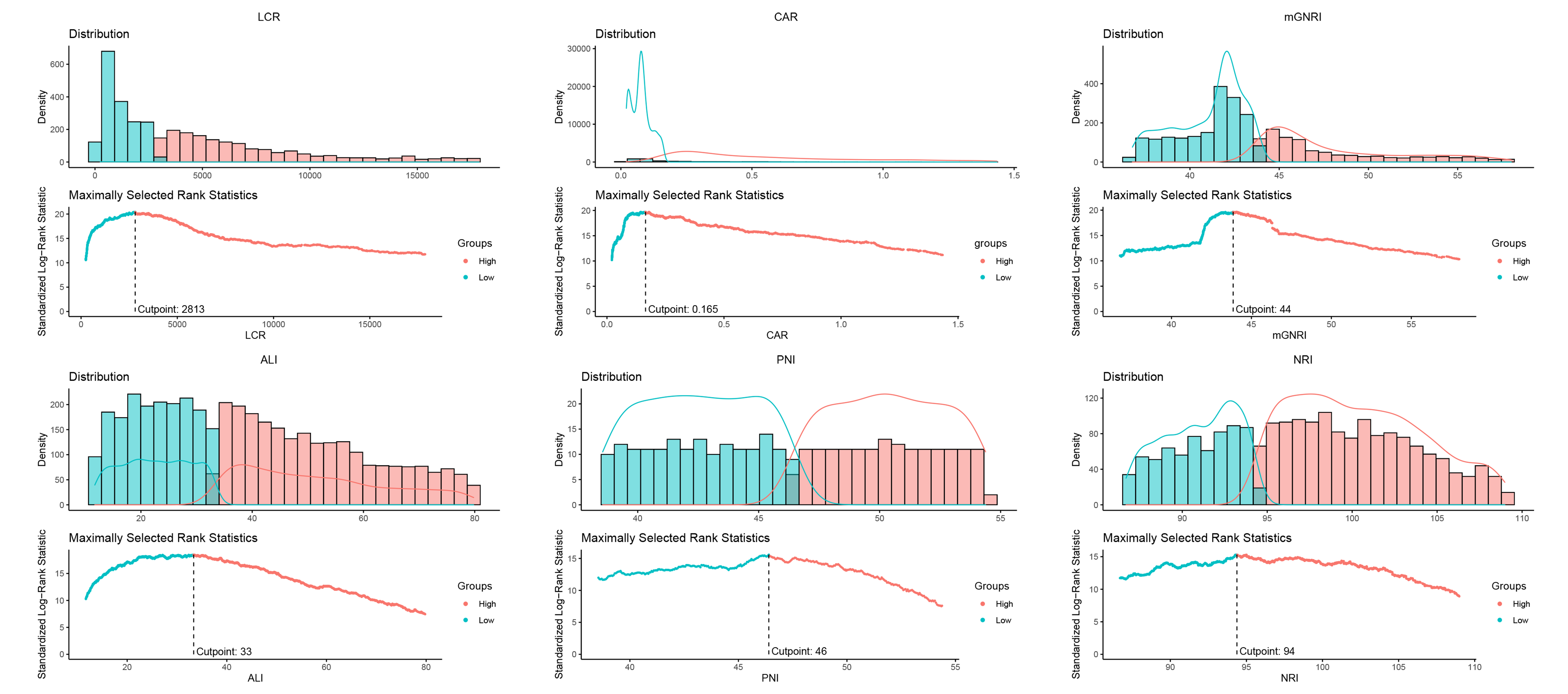


**Figure. S4.** Study design.


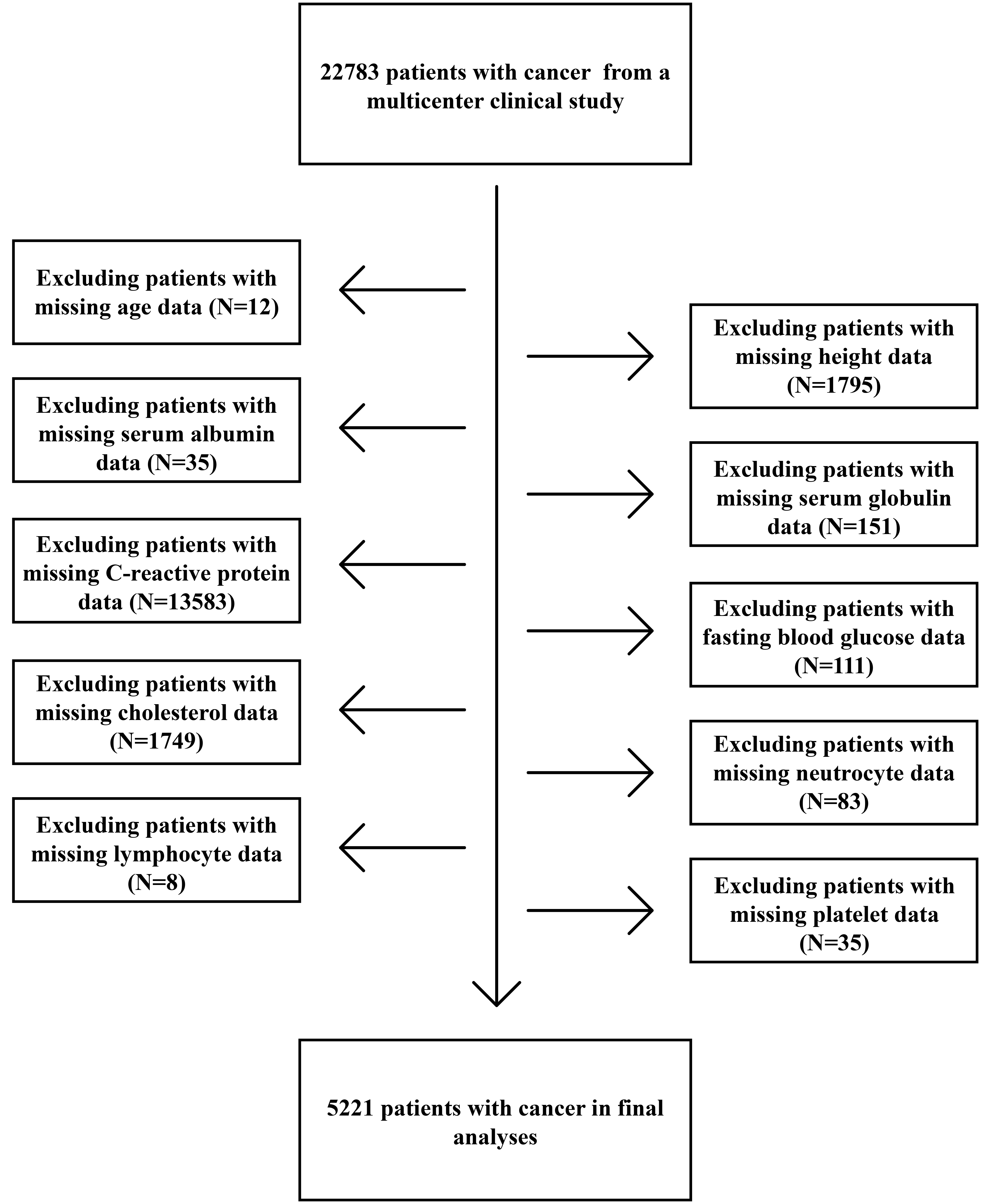


**Figure. S5.** The proportion of tumor stages and tumor types among different INS groups.


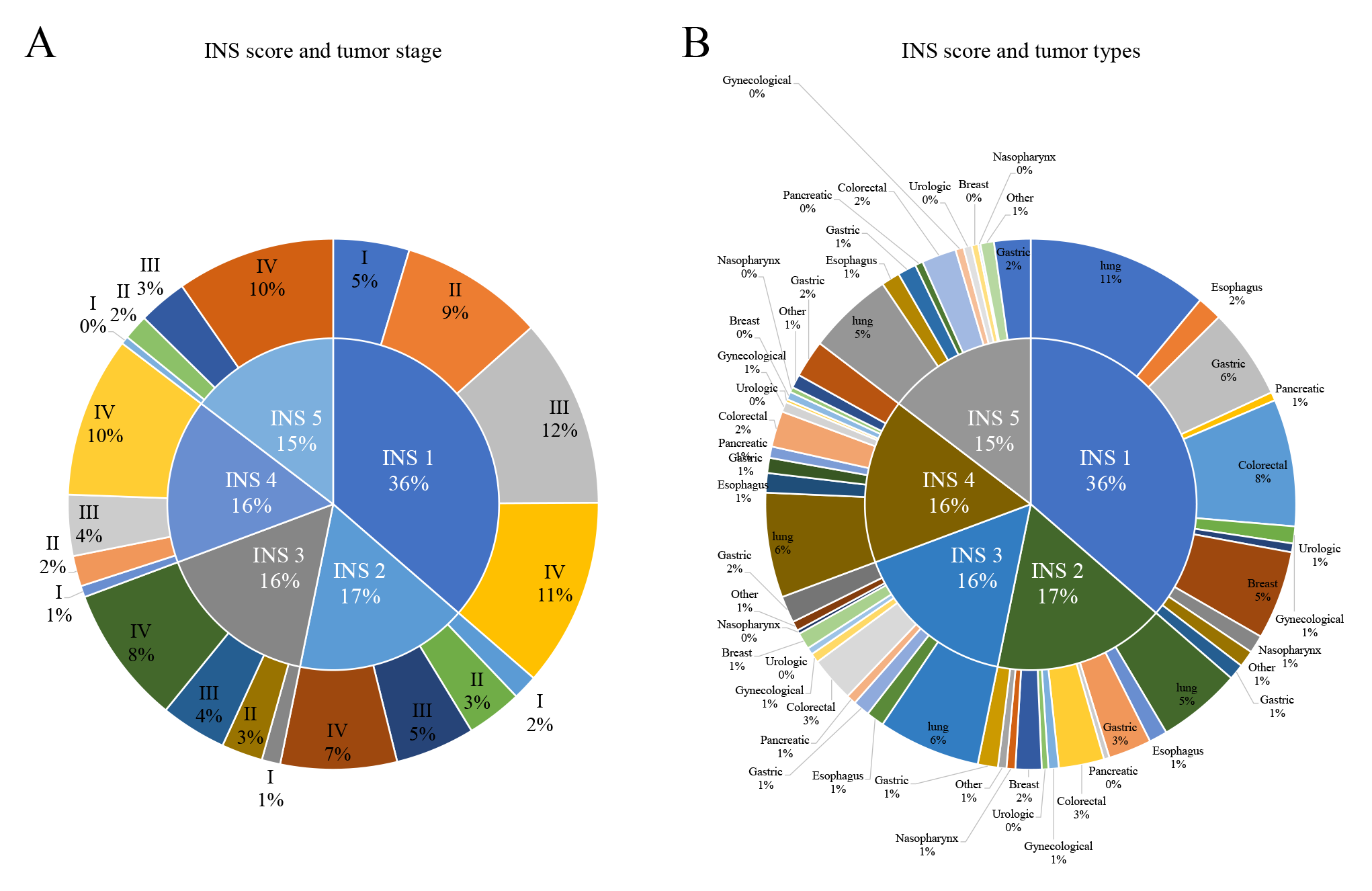


**Figure. S6.** Kaplan-Meier curve of systemic inflammation-related and nutrition-related markers in patients with cancer.


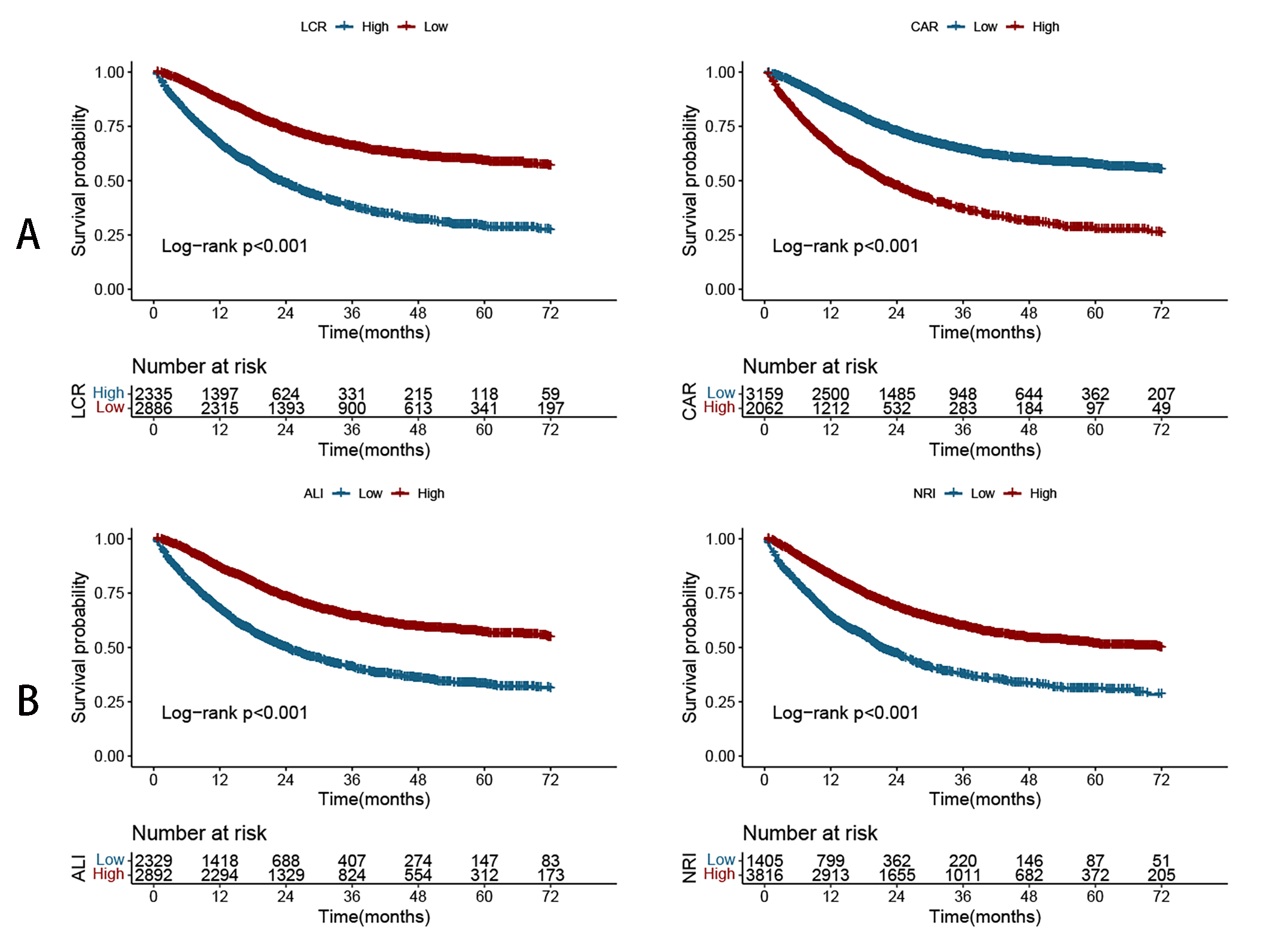


**Notes:** A, Systemic inflammation-related markers; B, Nutrition-related markers

**Figure. S7.** Stratified survival analysis of systemic inflammation-related and nutrition-related markers based on pathological stages.


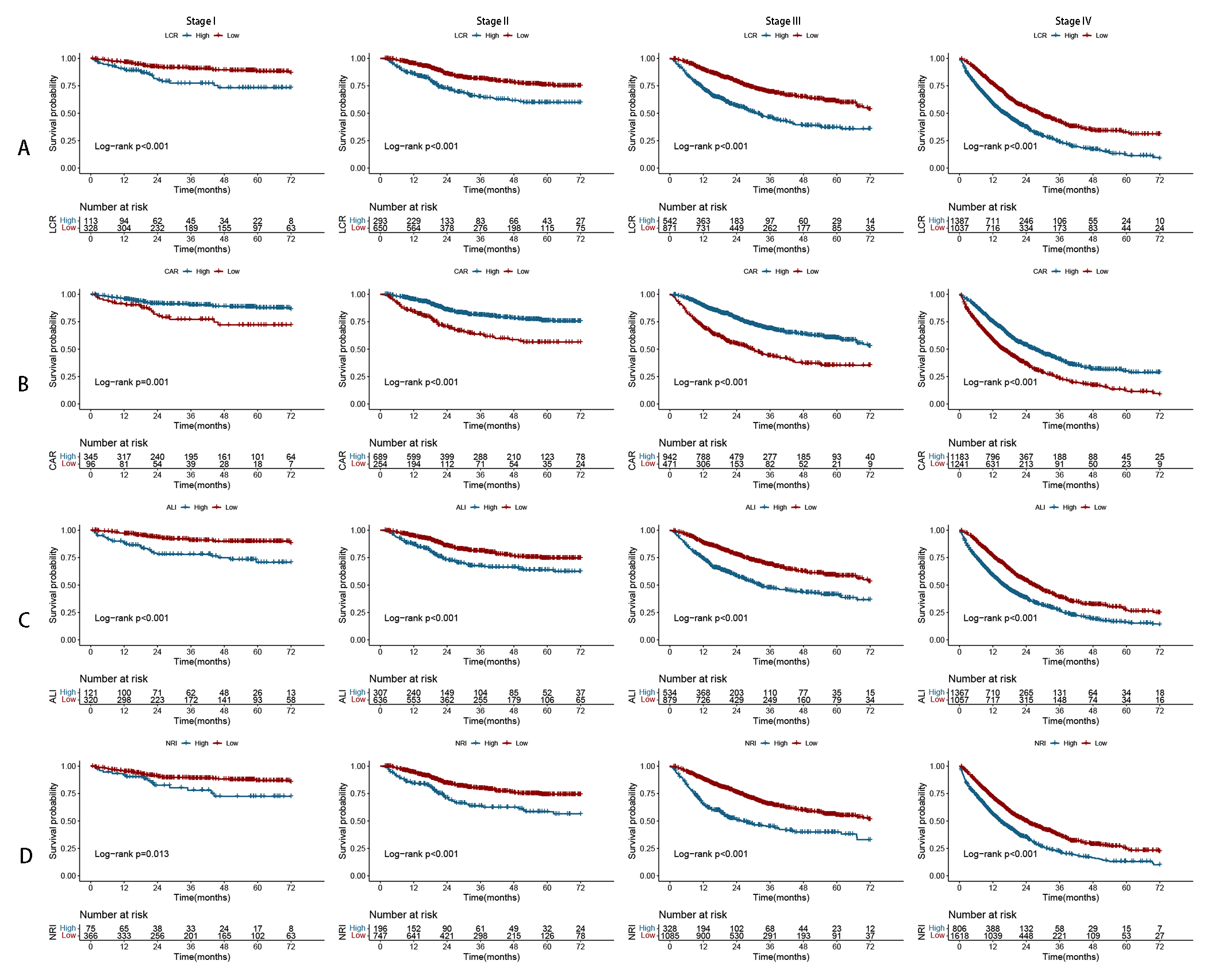


**Notes:** A, LCR; B, CAR; C, ALI; D, NRI

**Figure. S8.** Kaplan-Meier survival curve of inflammation-malnutrition biomarker score based different tumor types and pathological stages.


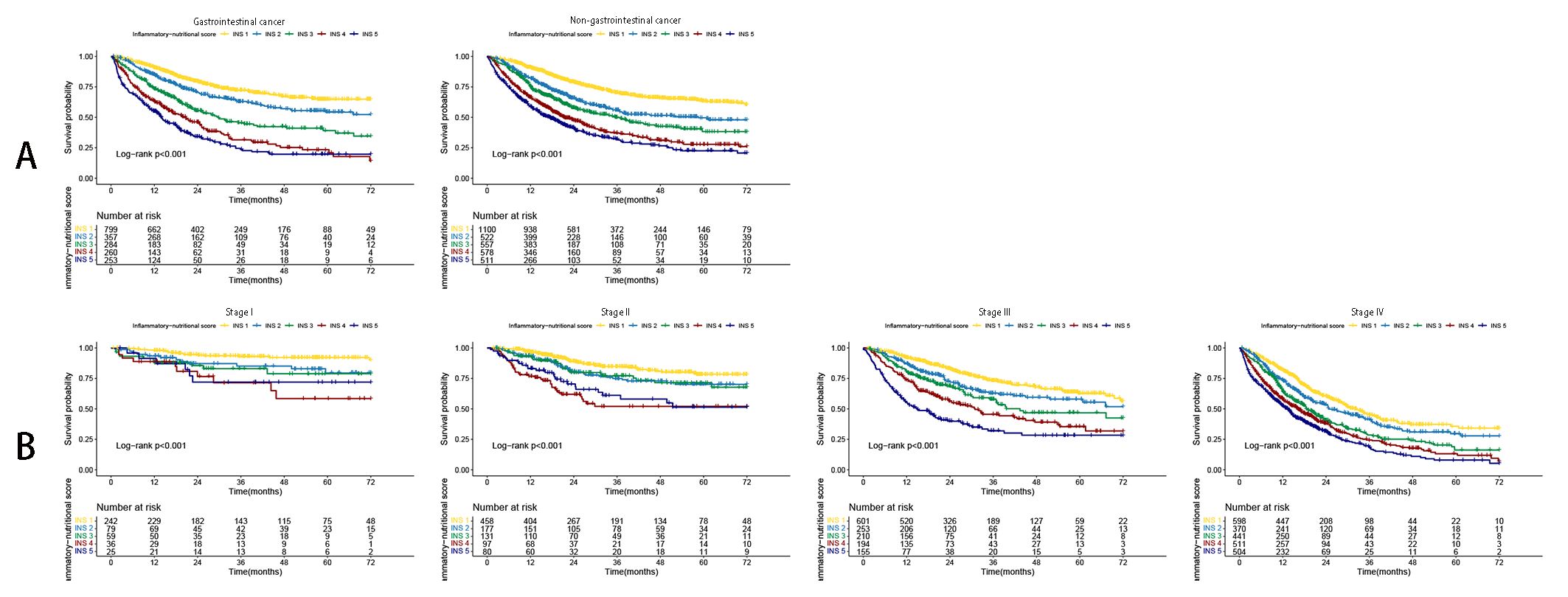


**Notes**: A, Kaplan-Meier survival curve of gastrointestinal cancer and non-gastrointestinal cancer; B, Kaplan-Meier survival curve of pathological stages I-IV.

**Figure. S9.** The association between systemic inflammation-related and nutrition-related markers and all-cause mortality in patients with cancer.


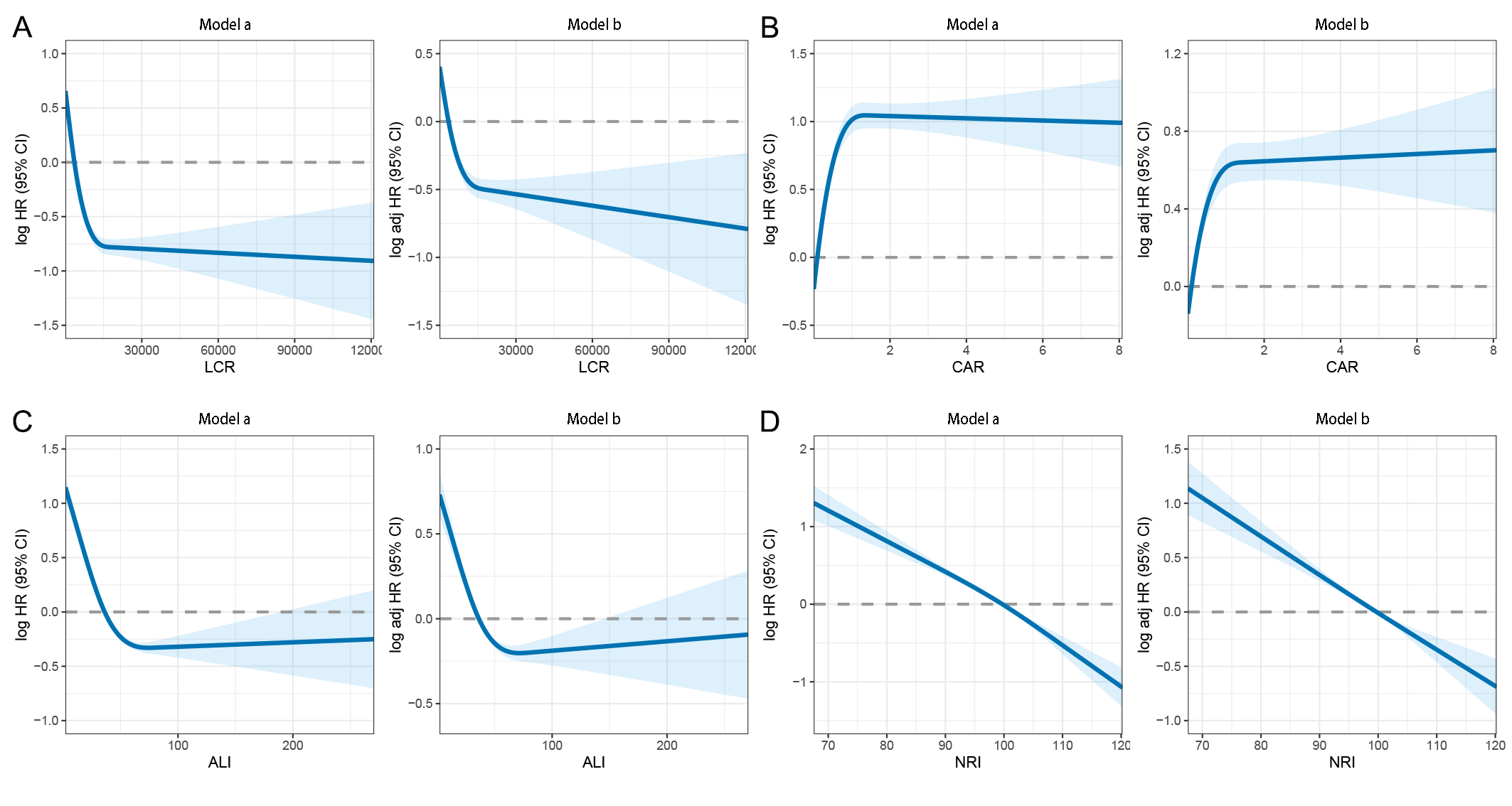


**Notes:** A, LCR; B, CAR; C, ALI; D, NRI

**Figure. S10.** The association between systemic inflammation-related and nutrition-related markers and hazard risk of overall survival in various subgroups.


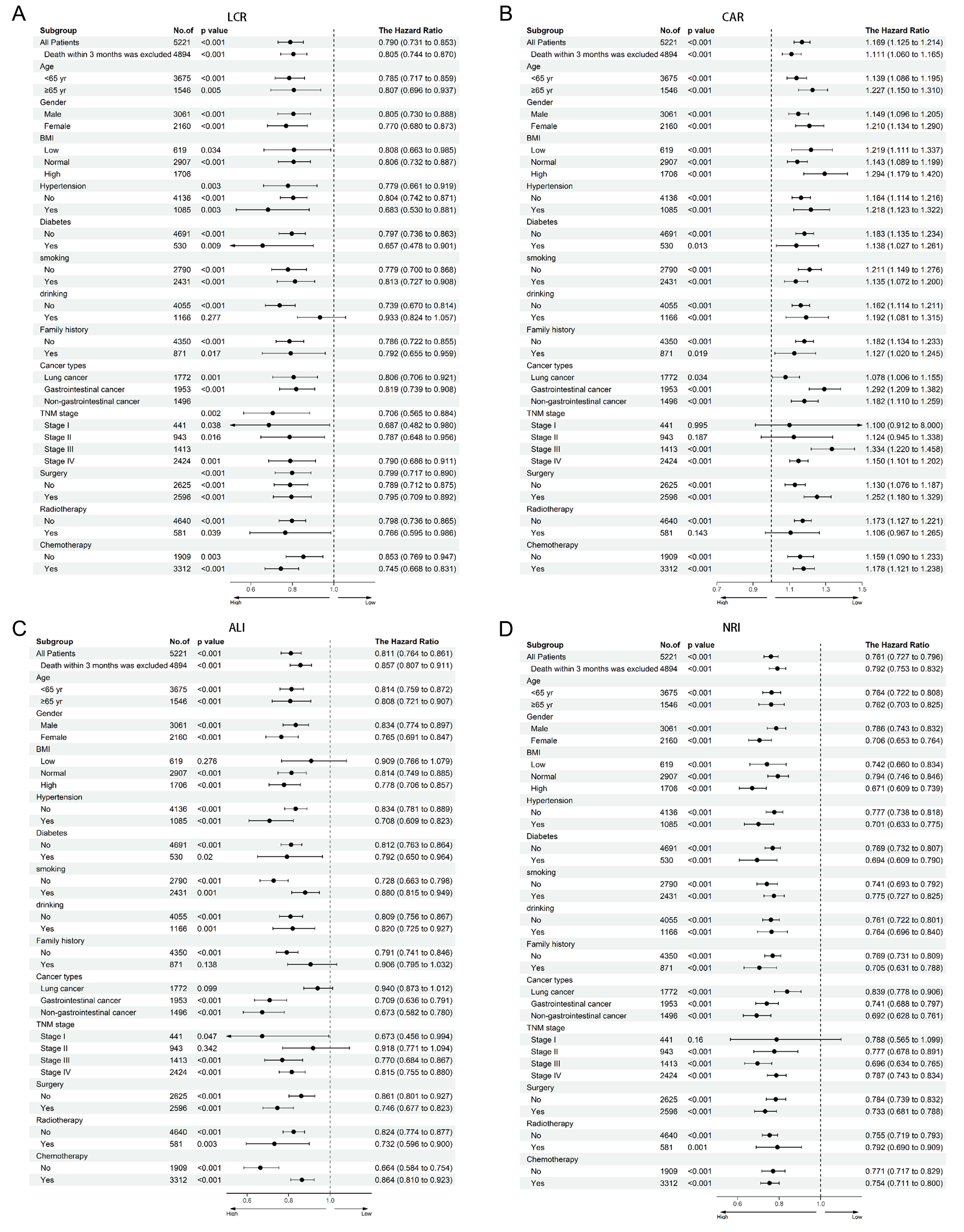


**Notes:** A, LCR; B, CAR; C, ALI; D, NRI. The model was adjusted for age, sex, BMI, TNM stage, tumor type, surgery, radiotherapy, chemotherapy, hypertension, diabetes, smoking, drinking, and family history.

**Figure. S11.** Dose-response effects of inflammatory-nutritional score based on subgroup.


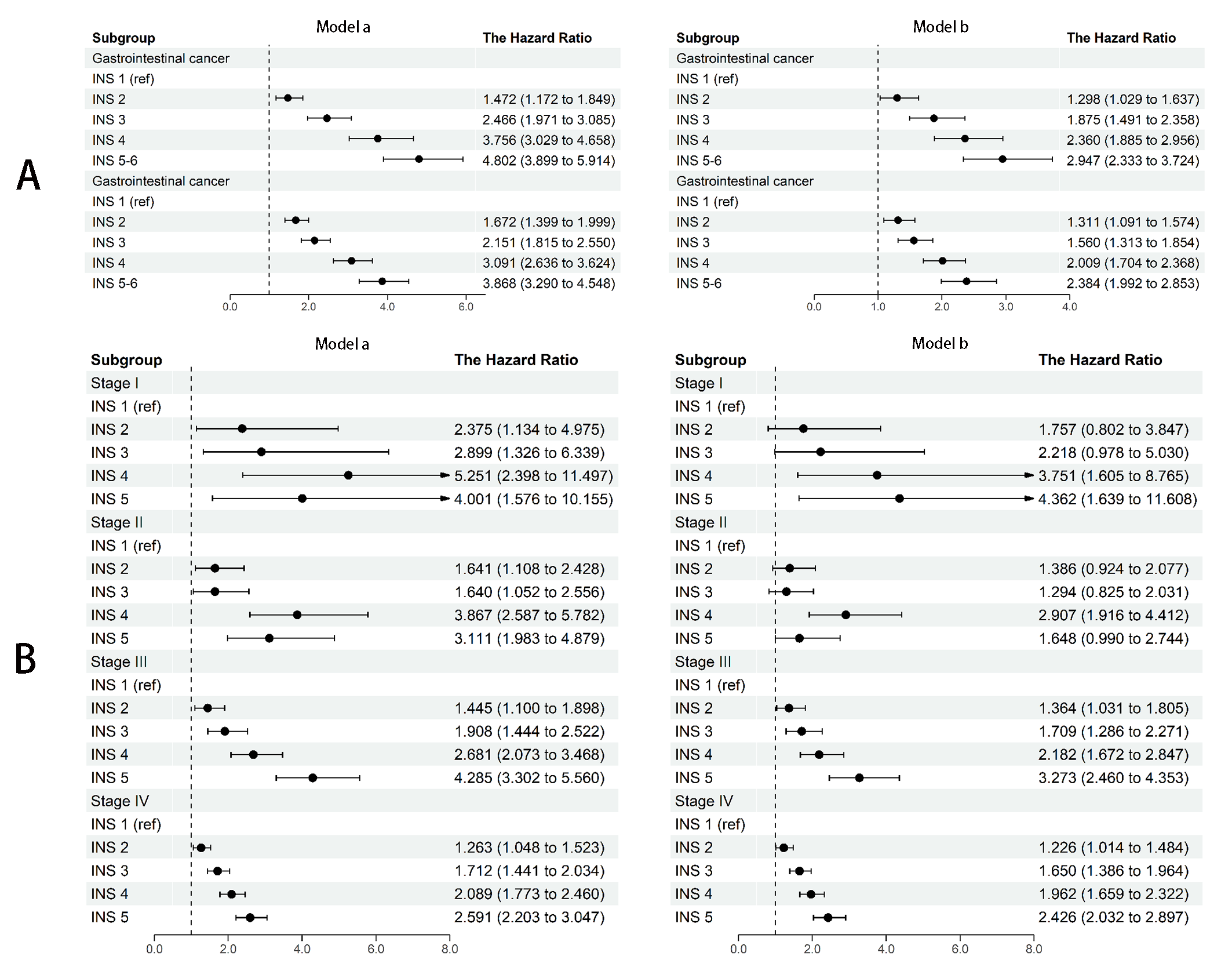


**Notes：**A, cancer types; B, pathological stage.

Model a: adjusted for age, sex, BMI, and TNM stage.

Model b: adjusted for age, sex, BMI, TNM stage, cancer types, surgery, radiotherapy, chemotherapy, hypertension, diabetes, smoking, drinking, and family history.
